# Nanoporosity boosts irradiation-induced dynamics in silica

**DOI:** 10.1039/d6ma00606j

**Published:** 2026-06-23

**Authors:** Francesco Dallari, Alessandro Martinelli, Jacopo C. Baglioni, Yuriy Chushkin, Giulio Monaco

**Affiliations:** a Dipartimento di Fisica e Astronomia “Galileo Galilei”, University of Padova via F. Marzolo 8 35131 Padova Italy francesco.dallari@unipd.it giulio.monaco@unipd.it; b Laboratoire Charles Coulomb Université de Montpellier Place Eugéne bataillon – cc 069 34090 Montpellier France; c European Synchrotron Radiation Facility – 71 Avenue des Martyrs, CS 40220 38043 Grenoble Cedex 9 France

## Abstract

Hard X-ray irradiation can activate relaxations in glasses even at temperatures well below their glass transition temperature, yet the nature of the involved structural rearrangements remains poorly understood. Here we compare bulk amorphous SiO_2_ with nanoporous silica to test how porosity and internal surfaces affect the radiation-induced structural and dynamical changes. In nanoporous silica, the intensity of the characteristic SiO_2_ first sharp diffraction peak is reduced by irradiation as in the bulk counterpart, but on a time scale more than an order of magnitude faster. Despite this accelerated structural evolution, the induced atomic-scale dynamics in nanoporous silica remains predominantly ballistic-like and displays reproducible intermittent fluctuations. At the first sharp diffraction peak, both materials show compressed-exponential relaxations, with markedly shorter time scales and stronger fluctuations in presence of nanostructures. These results identify internal interfaces as a key factor controlling hard-X-ray-driven relaxation in nanoporous silica, suggesting a practical route to tune radiation sensitivity in nanostructured glasses.

## Abstract 2

Hard X-rays induce ballistic-like dynamics in nanoporous silica, with 10 times faster dynamics than bulk SiO_2_. This points to surface-dominated phototoinduced mechanisms, crucial for predicting radiation-driven glass evolution.

## Graphical abstract

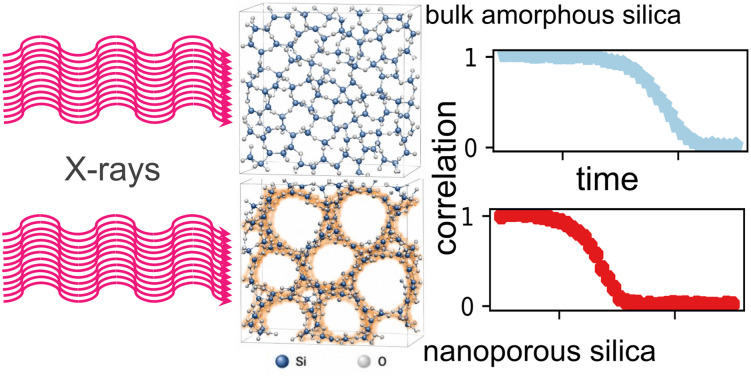

## Introduction

Oxide glasses are typically brittle materials. Compared to their softer counterparts, such as colloids and other soft matter systems, they exhibit significantly higher elastic moduli, which enable their use in mechanically demanding applications. This mechanical rigidity, however, comes at the cost of pronounced brittleness, as these materials tend to fracture upon relatively small deformations. However, under specific conditions it is nevertheless possible to induce plastic-like behaviour even in oxide and chalcogenide glasses. This can be achieved exposing these glasses to high doses of ionising radiation. For instance, in silica, keV electron irradiation has been shown to induce a response akin to that of a soft gel,^1–3^ while sub-bandgap irradiation in chalcogenide glasses can trigger non-thermal, light-induced fluidisation processes.^4^ These phenomena can be effectively probed using X-ray-based techniques and, in particular, X-ray photon correlation spectroscopy (XPCS), which allows to simultaneously trigger and probe the structural modifications. Experimental observations reveal several distinctive features, including the uniqueness of the structural and enthalpic state reached by a long enough irradiation, the coexistence of intrinsic and radiation-induced dynamics, an enhanced transport, and a dose-dependent evolution of the dynamical properties consistent with the existence of a yielding-like transition.^5,6^ So far, most investigations have focused on bulk systems. In this work, we extend them to nanostructured materials, specifically nanoporous silica, with the aim of exploring how nanoscale structures influence the material response to ionizing radiation in comparison to the behaviour of bulk SiO_2_ glass.

Nanoporous silica glass, commonly known as vycor, is a model disordered material characterised by a rigid, three-dimensional silica network permeated by an interconnected system of nanoscale pores. It is typically produced through phase separation in alkali–borosilicate glasses, followed by selective chemical leaching of the soluble phase, resulting in a high-purity amorphous SiO_2_ matrix with a well-defined porosity.^7^ The pore structure is bicontinuous, with characteristic pore diameters of the order of a few nanometers (∼2–10 nm), high internal surface area, and narrow pore size distribution. Due to its structural homogeneity at the nanoscale and chemical stability, nanoporous silica has become a prototypical system for studying confinement effects in liquids, phase transitions under nanoscale restriction, and transport phenomena in porous media. The interplay between the disordered silica matrix and confined phases leads to modified thermodynamic and dynamical behaviour, making nanoporous silica an important platform in fields ranging from glass physics and soft matter to nanofluidics and adsorption science. For these reasons, it is important to characterize and then understand how ionizing radiation can influence this class of materials.

## Experimental

The experiment was carried out at the ID10-COH beamline of the ESRF. The setup comprised two detectors: a CdTe Eiger 4M placed at 5.05 m from the sample for the XPCS measurements and a Pilatus 300 K placed at 49 cm from the sample (fixed position) to record a wider portion of the scattered intensity, *I*(*q*). The Pilatus geometry was calibrated using a LaB_6_ powder. The Eiger detector was mounted on a translation stage to vary the scattering angle *θ*, allowing us to span the *q*-range for XPCS between 0.6 Å^−1^ and 1.8 Å^−1^, where *q* = (4π/*λ*)sin(*θ*/2) is the exchanged wave-number and *λ* = 1.43 Å the wavelength of the X-ray beam. All measurements were performed with the sample in air, and an evacuated flight tube covered the last 4 m of the scattered beam path to reduce spurious air scattering. The photon flux was 3.7 × 10^12^ ph per s at 200 mA. The beam size at the sample was 6.8 µm × 5.0 µm FWHM (*H* × *V*).

Nanoporous silica samples (purchased from Boraglas GmbH) were provided as 5 mm × 5 mm plates, 0.5 mm in thickness, with a nominal pore size of 20 nm and a nominal volume ratio of silica/voids of 1. The plates were polished into wedges with a minimum thickness of ∼20 µm. The samples were mounted with the 5 mm × 5 mm base in the vertical plane oriented so that vertical translations probed lines of approximately constant thickness while horizontal translations selected the sample thickness. The local thickness was estimated from the transmitted X-ray intensity measured with a diode, assuming the nominal density provided by the supplier (1.11 g cm^−3^). The measured positions on the nanoporous silica sample were selected as a compromise between signal level and speckle contrast, given that the signal increases but the contrast decreases for thicker portions of the sample. Measurements were performed along two vertical lines corresponding to thicknesses of ∼40 µm and ∼70 µm.

Bulk silica was obtained from a capillary provided by Hilgenberg GmbH. The position for XPCS measurements on the capillary was selected to match the transmissivity of the vycor measurements, corresponding to a bulk-silica thickness of ∼30 µm. In both materials the thickness was well below the X-ray absorption length (160 µm for bulk, ∼320 µm for nanoporous silica), placing the experiment in a regime where the dose rate is effectively thickness-independent.

For each *q* value, ten repeated XPCS measurements were acquired for both nanoporous and bulk silica; between successive repetitions, the sample was translated vertically by 50 µm to reach non-irradiated (fresh) spots.

The WAXS scattered intensity, *I*(*q*), was processed using the PyFAI suite.^8^ The XPCS data reduction was performed first using the beamline software and later with a pipeline developed in-house. XPCS is a coherence-based technique in which time-resolved speckle patterns originating from density fluctuations in the sample are collected. Here we provide a small summary of the most important aspects of this technique, more detailed accounts can be found in, *e.g.* ref. 9–11.

Two-time correlation functions were computed for each probed *q* selecting the corresponding ROIs on the detector as:
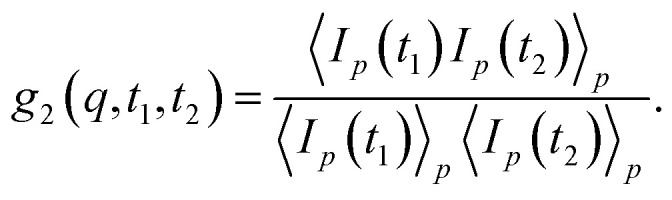
Here 〈⋯〉_*p*_ denotes the average over the pixels *p* in the ROI corresponding to the *q* of interest, and *I*_*p*_(*t*_1,2_) is the intensity recorded by pixel *p* at time *t*_1,2_.

The intensity correlation function is related to the intermediate scattering function *f*_*q*_(*t*_1_,*t*_2_) through the Siegert relation, *g*_2_(*q*,*t*_1_,*t*_2_) = 1 + |*f*_*q*_(*t*_1_,*t*_2_)|^2^. In presence of stationary dynamics, the autocorrelation function depends solely on the lag time *t* = |*t*_1_ − *t*_2_|, and the dynamics can be described by the simpler function *g*_2_(*q*,*t*). However, the main advantage of the two-time representation is that it captures non-stationary dynamics in evolving, out-of-equilibrium systems. In this case, which is relevant for the present experiment, the dynamics can be probed as a function of the irradiation time, which plays the role of the “waiting time” in the Physics of glassess. Several conventions exist for defining the waiting time *t*_w_ in two-time analyses.^12,13^ Here we use *t*_w_ = (*t*_1_ + *t*_2_)/2, which reduces sensitivity of the results at very short *t*_w_ but minimizes distortions of the shape of the relaxation function in the presence of strong fluctuations.^13^

For each value of *t*_w_, the thus obtained |*f*_*q*_(*t*)| function is typically described by a monotonous decreasing function, and a common choice is to use the Kohlrausch–Williams–Watts (KWW) function |*f*_*q*_(*t*)| = exp(−(*Γ*(*q*)*t*)^*β*^), where the relaxation rate *Γ*(*q*) and the exponent *β* depend on the waiting time *t*_w_. It is always possible to associate to the relaxation rate a characteristic time defined as *τ*(*q*) = 1/*Γ*(*q*). The information carried by these two independent parameters (*β* and *Γ* or *τ*) allows us to identify the dynamical regime in the sample. For example, *β* > 1 (“compressed” exponential) and a linear dependence of *Γ*(*q*) on *q* are indications of ballistic motion.^14^ In contrast, *β* < 1 (“stretched” exponential) indicates heterogeneities in the scattering volume and is observed in undercooled liquids.^15^ The KWW is in fact able to describe reasonably well a large group of monotonic relaxation processes offering a simple tool to evaluate their characteristic times.

## Results and discussion

**Fig. 1:**
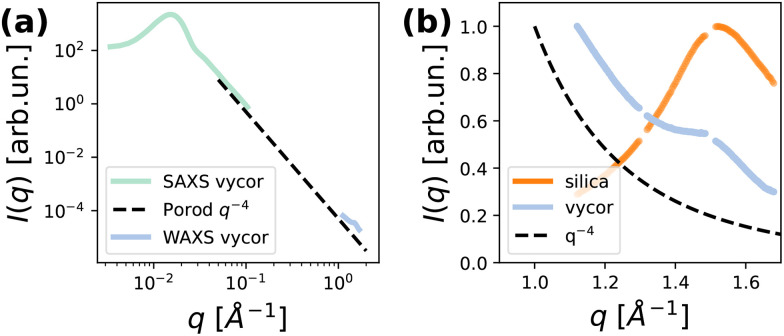
(a) log–log plot of the scattered intensity *I*(*q*) in the SAXS (green) and WAXS (orange) regimes. The black dashed line shows the Porod's law (*I* ∝ *q*^−4^) extrapolated from the high-*q* limit of the SAXS curve. The SAXS data were measured in a separate experiment on a pristine sample from the same batch. (b) Zoom of the signal in the WAXS regime: vycor (blue) and the extrapolated Porod contribution (black dashed) are compared with bulk silica (orange). Both curves are corrected by the background air scattering and normalized at their maximum value.

Nanoporous silica is often used as a calibration sample in small-angle X-ray scattering (SAXS) XPCS experiments. In fact, thanks to the large void fraction, it is an efficient X-ray scatterer, and the relatively narrow pore size distribution produces a characteristic *I*(*q*) profile (green curve in Fig. 1a), similar to that of other disordered systems such as colloidal glasses, with a broad maximum associated with the pore size.^7,16^ At higher *q*, the intensity rapidly decreases following the *q*^−4^ Porod regime (black dashed line), which is indicative of sharp interfaces and remains evident up to the interatomic length scale. At higher *q* ∼ 1.5 Å^−1^, nanoporous silica shows a second local maximum that recalls the well-known first-sharp diffraction peak (FSDP) of bulk silica.^17^ Notably, the two maxima at low- and high-*q* differ in intensity by ∼7 orders of magnitude.

When comparing bulk and nanostructured silica at large scattering angles (Fig. 1b), it is clear that both materials share the same atomic structure, yet their scattering profiles differ markedly. Bulk silica exhibits the well-known FSDP at ∼1.5 Å^−1^ (orange points), while in vycor only a weak local maximum is observed at the same *q* (blue points), with the scattered intensity still dominated by the Porod's contribution. This indicates that, even at the interatomic length scale, the signal measured in vycor predominantly arises from the interfaces between the pores and the silica matrix.

**Fig. 2:**
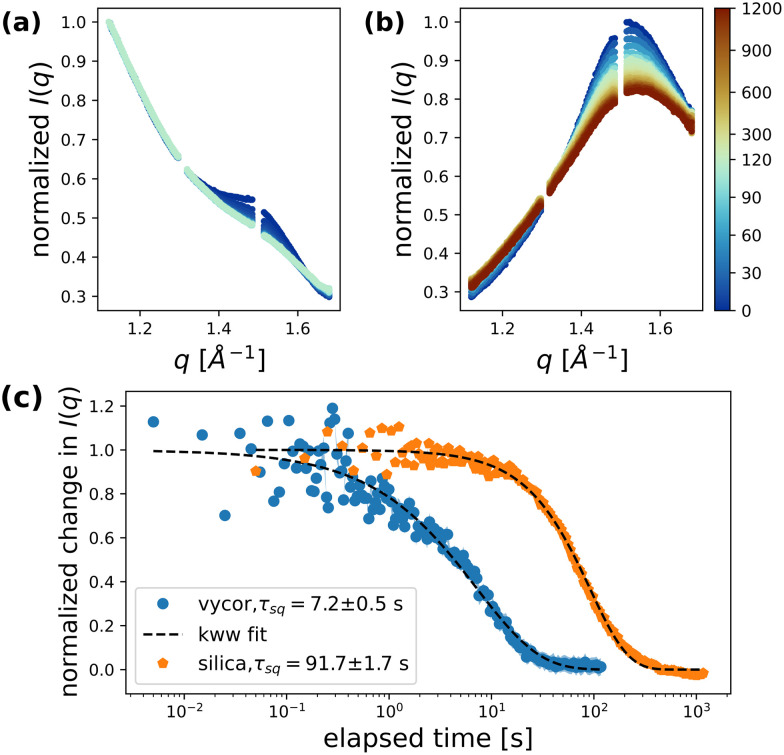
Irradiation-time (dose) evolution of the intensity profile for nanoporous (a) and bulk (b) silica. The two plots share the same colour scale and it's clear that the irradiation-induced changes take place on a shorter timescale for vycor. The changes of the peak height are displayed in panel (c) where it is possible to see that while nanoporous SiO_2_ (blue circles) reaches a seemingly stationary state with characteristic time of 7.2 ± 0.5 s, bulk SiO_2_ (orange pentagrams) requires 91.7 ± 1.7 s.

Ionising radiation is known to modify the atomic structure and dynamics of disordered materials at sufficiently large doses.^5,18,19^ This behaviour is also observed in nanoporous silica (Fig. 2a), where the FSDP characteristic of SiO_2_ decreases with dose and reaches an apparently stationary state after ∼20 s, becoming barely distinguishable from the underlying *q*^−4^ contribution. It must be pointed out that irradiation changes also the low-*q* scattering profile and slightly changes, in particular, the exponent of the Porod's regime.^16^ Even small changes in this exponent can greatly affect the WAXS regime of vycor, making precise assessments of the position and width of the corresponding FSDP difficult. Bulk SiO_2_ exhibits a similar evolution (Fig. 2b), but on a substantially longer time (dose) scale. To quantify this difference, we track the height of the local maximum in nanoporous and bulk silica normalised to the synchrotron current following the approach used *e.g.* in ref. 6. The resulting data (Fig. 2c) were rescaled to a common interval (from 1 to 0) and fitted using a KWW function. Although this model does not fully capture the shape of the obtained curves, in particular for silica, it provides an estimate of the characteristic time for the process. We obtain *τ*_sq_ = 7.2 ± 0.5 s for vycor and *τ*_sq_ = 91.7 ± 1.7 s for bulk silica, *i.e.*, more than an order of magnitude slower decay time in silica. The corresponding values for the stretching coefficients are 0.71 ± 0.05 and 1.17 ± 0.03 for vycor and silica, respectively.

The reduction in intensity of the FSDP with irradiation time (dose) is accompanied by a broadening and a shift towards larger *q*'s of the FSDP, similarly to what has been observed also in previous experiments^18^ and in other oxide glasses.^5^ Similar changes are present in the nanoporous sample, albeit less visibly. The intensity reduction, broadening and shift towards higher *q*'s of the FSDP are associated with the densification of the material and we know from previous studies that this density change is reflected in the evolution with the dose of the structural dynamics,^5^ setting a kinetic time-scale that separates the initial ballistic-like regime from the final stationary, liquid-like one.

**Fig. 3:**
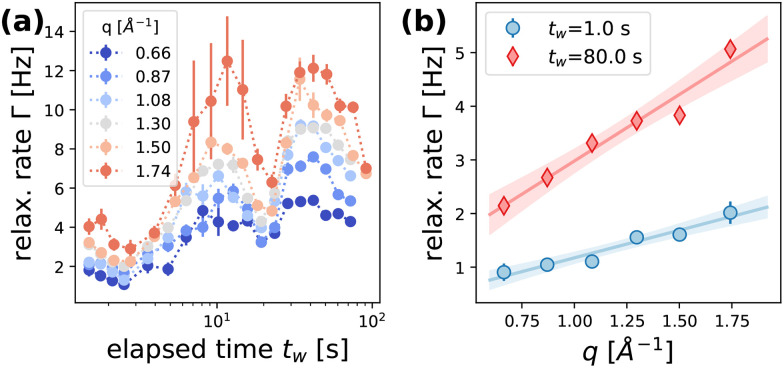
(a) Irradiation-time (dose) evolution of the XPCS relaxation rates of nanoporous silica at different *q*'s. The relaxation rate *Γ*(*q*, *t*_w_) displays several fluctuations that can be seen simultaneously at all *q*'s. (b) *q*-Dependence of the relaxation rates for nanoporous silica irradiated for ∼1.0 s (cyan circles) and 80 s (red diamonds). The lines are linear fits and the shaded areas are confidence bands. The intercepts of the linear trends remain compatible with zero within two standard deviations in all cases.

Based on previous studies in lithium borate^5^ and chalcogenide^6^ glasses, the observed approach to a stationary state in terms of structure is expected to signal that both vycor and silica approach a yielding state. In terms of dynamics, this is marked by a stationary state with a stretched dynamics that follows an initial transient with ballistic-like dynamics. In the yielding state, the dynamics is also characterized by a weak *q*-dependence and a visible de Gennes narrowing effect (*i.e.* a slowing down of the dynamics in correspondence to the peak of the FSDP).^5^ However, when the dynamics measured in vycor by XPCS are examined, it is clear that this is not exactly the case. Fig. 3(a) displays the relaxation rate as a function of the irradiation time for *q*-values ranging from 0.66 Å^−1^ to 1.74 Å^−1^. While there is a general trend towards faster relaxations, there are also strong fluctuations at all length-scales. It is interesting to note that such events are a consequence of the irradiation and not of the heterogeneous nature of the sample. In fact, the measurements at each *q*-value are the result of the average of 10 repetitions on different spots, and the fluctuations appear in all repetitions at the same irradiation times, meaning that these are clearly reproducible features. While we cannot provide at this stage a definite interpretation of this effect, we can observe that these fluctuations shows up at the same time at all probed *q*'s, meaning that they probably reflect density changes in vycor. These density changes might take place in successive steps possibly involving the collapse of the pores as well as the densification of the bulk as in silica. This hypothesis would however require a dedicated beamtime for verification.

In Fig. 3(b) the *q*-dependence of the relaxation rate is plotted for irradiation times of 1.0 s and 80 s, this latter time corresponding to a condition where the structure of vycor has already reached the stationary state. It is possible to see that in both cases *Γ* ∝ *q*, meaning that we are still in a ballistic regime despite the fact that the structure appears to have reached a stationary state. This is different from what we have previously observed in irradiated bulk glasses. An interesting detail is that the largest outlier of the 80 s irradiated dataset is the point at *q* = 1.5 Å^−1^ which corresponds to the position of the FSDP. The low-rate value observed there could be an indication of the expected de Gennes narrowing effect, though lying on top of a linear *q*-dependence. However, this observation would require more data and a more refined analysis to be confirmed.

**Fig. 4:**
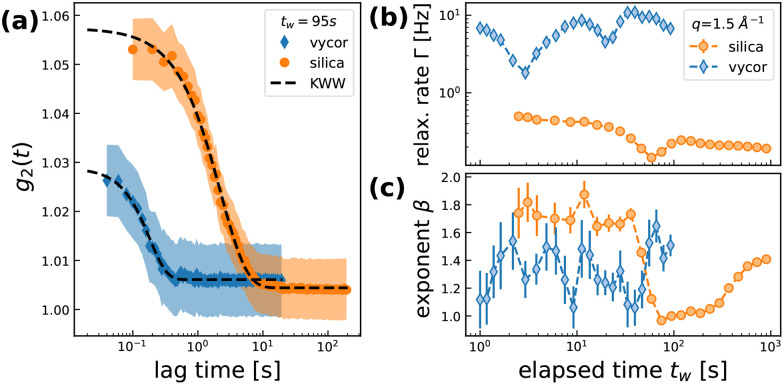
Comparison of the observed dynamics at the FSDP in nanoporous and bulk silica. (a) *g*_2_(*q*_max_, *t*) measured at 95 s of irradiation. Vycor (blue diamonds) displays a clearly faster dynamics than silica (orange circles). The black dashed lines are a KWW fit to the data. In (b) and (c) the relaxation rates *Γ* and shape parameters *β* are reported for both materials, see legend. While vycor (blue points) displays large fluctuations in *Γ* and *β*, SiO_2_ shows only one of such events.

Focusing our attention on the FSDP at high *q*, we can compare the characteristics of the induced dynamics in vycor with those of silica. In Fig. 4(a) we can see the *g*_2_(*q*,*t*) function of nanoporous and bulk silica at 95 s of irradiation. They can both be described by a compressed exponential with the main differences being the characteristic time and contrast, both clearly smaller in the case of nanoporous silica. The difference in contrast is a direct consequence of the larger thickness of the nanoporous sample in comparison to the bulk silica one.^20–22^ The difference in relaxation time corresponds to a ∼10 times faster timescale in vycor than in bulk SiO_2_, similar to what previously observed in terms of structure. Moreover, the irradiation-time dependence for the two materials is also different. As it can be seen in Fig. 4(b), silica tends to monotonically slow down its relaxation rate with increasing dose, except for a single fluctuation; vycor tends instead to speed up and shows more fluctuations, as also seen in Fig. 3(a). The shape parameter *β*, reported in Fig. 4(c), remains compressed in both materials. In silica, it remains stable up to irradiation times associated to the appearance of the fluctuation in *Γ*(*t*_w_), where it drops, reaching values close to 1. In vycor, the behaviour is more jagged. These changes in *β* can be partially explained by relatively sharp fluctuations in *Γ* within the time interval used to compute *g*_2_(*t*). Notably, previous studies in other glasses^5,6^ showed that at the yielding condition, the stretching coefficient decreases to a value well below 1, and *Γ* (or equivalently *τ*) becomes almost *q* independent with the exception of a modulation in correspondence to the peaks of the scattered intensity, similarly to what is usually observed in liquids (de Gennes narrowing effect). In liquids, this reflects the slowing down of the collective relaxation at length scales commensurate with the nearest-neighbour ordering: the structure at these preferred spatial distances is more “rigid” and the density fluctuations decay more slowly. The appearance of this feature is then a signal that the collective part of the intermediate scattering function is dominant in the probed dynamics. The fact that in silica the correlation functions are compressed at all explored irradiation times is an indication that while we have approached the stationary, yielding state, we did not fully reach it.

Both structural and dynamical data indicate that the characteristic time scales for the irradiation-induced changes in nanoporous silica are an order of magnitude shorter than in bulk amorphous SiO_2_, despite the identical composition. A possible explanation is suggested by Fig. 1, which shows that the scattered intensity from internal surfaces in vycor dominates the total intensity even at relatively large *q*'s. This observation is consistent with reports that surface-associated dynamics can be faster than bulk dynamics, due to reduced geometric constraints and lower effective activation barriers.^23–25^ Although the processes discussed here are driven by hard X-ray absorption, it is noteworthy to observe that thermally activated mechanisms in silica (*e.g.*, self-diffusion) display a comparable acceleration when comparing surface and bulk.^26^

Several additional differences between vycor and bulk silica may contribute to their distinct response to hard X-ray irradiation. First, vycor has a substantially lower thermal conductivity;^27^ at a fixed dose rate this can lead to higher local temperatures and steeper temperature gradients than in the bulk materials typically studied. Second, the nanostructure of vycor is expected to strongly affect how the dose is deposited in space. The dynamics observed here is triggered by defect creation, largely driven by secondary electrons generated by the primary photoelectrons. Because photoelectron tracks and the associated cascades can extend over length scales larger than the typical pore spacing,^28,29^ the energy deposition in vycor is less homogeneous, producing highly defected regions around the absorption sites.

We also note that ion-irradiation studies have shown that nanoporous silica can undergo pore shrinkage and densification of the SiO_2_ network,^30^ with densification also occurring in bulk SiO_2_.^31^ Changes in the pore size mainly affect the XPCS signal at the nanometer length scales, whereas at the interatomic distances probed in this study such structural evolution may instead be less critical. One last remark concerns the details of the observed relaxation functions. In nanoporous samples we should expect, in principle, the presence of two contributions, one from the surfaces and a weaker and slower one from the SiO_2_ framework. This would imply that additional relaxations could be present at large lag times, but to actually being able to observe them one would need to significantly improve the signal to noise ratio.

## Conclusions

We investigated the response to hard X-ray irradiation of two materials that share the same composition and the same local atomic structure (amorphous SiO_2_), but differ by the presence of nanoscale porosity. Both materials are static and stable at room temperature, yet they exhibit structural changes and a relatively fast dynamics under hard X-ray irradiation. Nanoporous silica shows irradiation-induced changes in the dynamics that have not been observed in previously studied bulk glasses: a ballistic-like dynamics and compressed correlation functions at irradiation-times well within the range where the material has reached a stationary response. The overall response of vycor (time-scales, fluctuations and dose-dependence) differs substantially from that of bulk silica. The most evident difference is that the time dependence for irradiation-induced effects in vycor is more than an order of magnitude faster than in silica. We have attributed this difference to the dominance of surface-related photo-induced effects in nanoporous silica. Interestingly, the same trend can be found in looking at thermally activated mechanisms on surfaces, suggesting a connection between the two phenomena.

## Author contributions

F. D.: conceptualization, software, formal analysis, writing – original draft; A. M.: investigation, data curation, writing – review and editing; J. C. B.: investigation, data curation, writing-review and editing; Y. C.: resources, writing – review and editing; G. M.: conceptualization, investigation, data curation, supervision, writing – review and editing, funding acquisition.

## Conflicts of interest

There are no conflicts to declare.

## Data Availability

The data for this article, including reduced datasets, will be available at Research Data Unipd, at https://doi.org/10.25430/researchdata.cab.unipd.it.00001844. Raw data are available at the ESRF data portal at https://doi.org/10.15151/ESRF-ES-1186977893.
